# Characterisation of gp34, a GPI-anchored protein expressed by schizonts of *Theileria parva* and *T. annulata*

**DOI:** 10.1016/j.molbiopara.2010.03.018

**Published:** 2010-08

**Authors:** Gondga Xue, Conrad von Schubert, Pascal Hermann, Martina Peyer, Regina Maushagen, Jacqueline Schmuckli-Maurer, Peter Bütikofer, Gordon Langsley, Dirk A.E. Dobbelaere

**Affiliations:** a Division of Molecular Pathobiology, DCR-VPH, Vetsuisse Faculty, University of Bern, CH-3013 Bern, Switzerland; b Institute of Biochemistry & Molecular Medicine, Medical Faculty, University of Bern, CH-3012 Bern, Switzerland; c Laboratory of Comparative Cell Biology of Apicomplexan Parasites, Département de Maladie Infectieuse, Institut Cochin, 75014 Paris, France

**Keywords:** GPI, Glycosylphosphatidylinositol, Plk1, Polo-like kinase1, PBD, polobox domain, Apicomplexa, Transformation, Mitosis, Cytokinesis, Intracellular parasite, Surface protein

## Abstract

Using bioinformatics tools, we searched the predicted *Theileria annulata* and *T. parva* proteomes for putative schizont surface proteins. This led to the identification of gp34, a GPI-anchored protein that is stage-specifically expressed by schizonts of both *Theileria* species and is downregulated upon induction of merogony. Transfection experiments in HeLa cells showed that the gp34 signal peptide and GPI anchor signal are also functional in higher eukaryotes. Epitope-tagged Tp-gp34, but not Ta-gp34, expressed in the cytosol of COS-7 cells was found to localise to the central spindle and midbody. Overexpression of Tp-gp34 and Ta-gp34 induced cytokinetic defects and resulted in accumulation of binucleated cells. These findings suggest that gp34 could contribute to important parasite–host interactions during host cell division.

## Graphical abstract

We characterised gp34 (yellow), a GPI-anchored protein that is expressed on the surface of the schizont (red) of the transforming intracellular parasite *Theileria*. The host cell nucleus is stained blue.

## 1 Introduction

The protozoan parasites *Theileria parva* and *T. annulata* are transmitted by ticks and cause severe lymphoproliferative diseases in cattle in large regions of Africa and Asia. Much of the pathology can be attributed to the fact that the intracellular schizont stages of these parasites are capable of transforming the leukocytes they infect, resulting in a rapid clonal expansion of the parasitized cell population. The schizont is strictly intracellular and differs from several other apicomplexan parasites such as *Plasmodium*, *Toxoplasma* or *Eimeria*, in that it is not enclosed in a parasitophorous vacuole [1]. Positioned in the cytosol, the schizont orchestrates the activation of an array of signalling pathways that promote host cell proliferation and protection against apoptosis (reviewed in [2,3]). Phenotypic analyses of *Theileria*-transformed cell lines also point towards interference with regulators of pro-inflammatory responses, which, in turn, are thought to contribute to the development of disease [4]. The interactions between the parasite and the host are complex, involving a range of different proteins, most of which are unknown. Members of the *T. annulata* TashAT family [5] and SuAT [6] have been reported to be released into the host cell cytosol and translocate to the nucleus, where they are hypothesized to interfere with host cell transcription. Another protein, called TaSE, was also reported to be secreted by the parasite and interact with host cell microtubules in a punctate manner [7]. Little is known about the repertoire of schizont surface proteins potentially involved in parasite–host cell interactions. Both *T. parva* and *T. annulata* express an immunodominant surface protein, designated polymorph immunodominant molecule (PIM) [8], or *T. annulata* surface protein (TaSP) [9], respectively. PIM is the major schizont antigen recognized by the sera of infected animals [10]. The protein shows unusual characteristics including extensive, variable QP-rich domains and its function is still unknown [11] although an interaction with microtubules has recently also been proposed [12]. Furthermore, it has been reported that antibodies raised against 11E, a secretory type glutaredoxin homologue also stain the schizont surface [13]. The availability of annotated *T. parva* and *T. annulata* genomes [14,15] opened up new opportunities to search for proteins predicted to be expressed on the schizont surface.

Using bioinformatics, we searched the *T. annulata* and *T. parva* annotated proteomes [14,15] for schizont proteins predicted to contain an N-terminal signal peptide and a C-terminal GPI anchor signal. In this work, we characterise a GPI-anchored protein, called gp34, which is conserved in both *T. parva* and *T. annulata* and expressed on the surface of the transforming schizont.

## 2 Materials and methods

### 2.1 Isolation of gp34, generation of expression constructs and antibodies

To identify potential GPI-anchored proteins expressed on the schizont surface, a Complex/Boolean query of *T. annulata* geneDB (http://old.genedb.org/genedb/annulata) was carried out using the search parameters: ‘Proteins containing a predicted GPI-anchor’ and ‘Proteins containing a predicted signal peptide’; only genes that were represented in the schizont EST library were further selected and those encoding proteins containing multiple membrane-spanning domains were not considered.

Coding regions of TA06510 and TP01_0939 were amplified by PCR from cDNA obtained from *T. annulata* (TaC12)-infected macrophages [16] and *T. parva* (Muguga)-infected T lymphocytes (TpM (D409)T4) [17]. Primers used for the isolation of the coding sequence excluding the regions encoding the signal peptide and GPI anchor signal were 5′-TGCGAATTCCAAAGCTTATTGAGGAGGATCTACG-3′, 5′-TGCCTCGAGAGTAACGAAACTTGATAATC-3′ for TA06510 and 5′-TGCAGATCTAAGCTTTCCTCGGGGAAGTCGGC-3′, 5′-TGCCTCGAGTCATCATAATCTGAAGGGGATTC-3′ for TP01_0939. PCR products were cloned into pGEX-6P vectors (GE Healthcare; digested with *Eco*RI–*Xho*I for TA06510 and *Bam*HI–*Xho*I for TP01_0939) using restriction sites introduced with the primers (*Eco*RI–*Xho*I for TA06510; *Bgl*II–*Xho*I for TP01_0939) and recombinant proteins expressed in *E. coli* BL21 Star (Invitrogen). Recombinant gp34 was purified using glutathione sepharose beads (GE Healthcare) and separated from GST using PreScission Protease (GE Healthcare). The purified protein was supplemented with GERBU adjuvant 10 (GERBU Biochemicals) and used to immunize rats (for anti-Ta-gp34 production) and rabbits (for anti-Tp-gp34). Antibodies were subjected to antigen-specific affinity purification as described [18].

To express epitope-tagged gp34 on the surface of mammalian cells, a pmaxCloning (Amaxa) expression plasmid was generated as follows: the *T. parva* gp34 precursor protein, the full coding sequence of TP01_0939 including the signal peptide, the mature protein, and the GPI anchor signal was amplified by PCR using the overlapping forward primers 5′-TGCAGATCTCGCCACCATGAAGTATATTTTATTTATTTTAATTTCAAC-3′ and 5′-TAATTTCAACTTGCGTGGTTTCCTCGGGGCCCGCCATGAAG-3′ as well as the reverse primer 5′-TGCCTCGAGTCATCAAAAGTTCATGAGTAAGAAAGCG-3′. The sequence encoding T7-QPRD1 of *T. parva* PIM was amplified from T7-QP-rd-His [11] by PCR and inserted downstream of the sequence encoding the signal peptide. The QPRD1 domain is recognized by an anti-PIM monoclonal MAb5 [11].

For the expression of Tp-gp34 in the cytoplasm of mammalian cells, part of the TP01_0939 coding sequence (representing aa 15–285 and lacking the signal peptide and GPI signal sequence) was amplified using the primers 5′-TGCAGATCTAAGCTTTCCTCGGGGAAGTCGGC-3′, 5′-TGCCTCGAGTAATCTGAAGGGGATTC-3′ and inserted into the pmaxCloning vector using *Bgl*II and *Xho*I restriction sites. A sequence encoding the C-terminal V5 epitope tag was amplified from pcDNA3.1-V5/His (Invitrogen) with the primers 5′-TGCCTCGAGGTCGACATCGATGGTAAGCCTATC-3′ and 5′-TGCGAGCTCATCGATCGTAGAATCGAG-3′ and inserted 3′ of the TP01_0939 insert via *Xho*I and *Sac*I restriction sites. Likewise, for the cytoplasmic expression of an unanchored version of Ta-gp34 in mammalian cells, the coding sequence of TA06510 representing aa 21–290 was cloned into pmaxCloning encoding the V5 tag after excision of the TP01_0939 sequence (see above) in a *Bgl*II–*Xho*I digest. The insert was generated by PCR using primers 5′-TGCGGATCCATTGAGGAGGATCTACG-3′ and 5′-TGCCTCGAGAGTAACGAAACTTGATAATC-3′ and digested *Bam*HI–*Xho*I.

To generate an N-terminally EGFP-tagged version of Tp-gp34, the TP01_0939 coding sequence representing the mature protein was excised from pGEX-6P–TP01_0939 (see above) in a *Bgl*II–*Xho*I digest and inserted into pEGFP-C1 (Clontech).

Plasmids used as control for the binucleation assay were pmaxGFP (Amaxa) and TP03_0882-ΔSP in pmaxCloning vector.

To generate truncation mutants of Tp-gp34, the TP01_0939 coding sequence was partially amplified by PCR using the primers 5′-TGCAGATCTAAGCTTATGGGGAAGTCGGCTCTGGAGG-3′ and 5′-CCGCTCGAGGGA TTTAGTTACGCTCC-3′ for aa 17–139, 5′-TGCAGATCTATGCTGACGAAGCTGGATGAAAG-3′ and 5′-TGCCTCGAGTAATCTGAAGGGGATTC-3′ for aa 140–285, or 5′-TGCAGATCTATGTATAAGGAATTCTATGG-3′ and 5′-TGCCTCGAGTACCGGGAACTCATCTATCGC-3′ for aa 81–220. For aa 17–285, the coding sequence was amplified using primers 5′-TGCAGATCTAAGCTTATGGGGAAGTCGGCTCTGGAGG-3′ and 5′-TGCCTCGAGTAATCTGAAGGGGATTC-3′. PCR products were inserted into the pmaxCloning vector encoding a C-terminal V5 tag (see above) using *Bgl*II/*Bam*HI and *Xho*I restriction sites.

Mutations of the Plk1 motifs in Tp-gp34 were generated by a two-step PCR. Using the TP01_0939 coding sequence as template, flanking primers and complementary mutagenic primers were used to generate two partially overlapping amplicons that were fused in a second PCR. The flanking primers used were 5′-TGCAGATCTAAGCTTATGGGGAAGTCGGCTCTGGAGG-3′ and 5′-TGCCTCGAGTAATCTGAAGGGGATTC-3′. The mutagenic primers were 5′-GCTAGGTTCAAGGAAGCCGCCAAGAAACACGCCGCC-3′ for S69A + S70A, 5′-CATATAAATTTGATGAAAGCGCCCCACTGTTTAGAAG-3′ for D104A, 5′-GAATCTCACTGTGTTTAGGAAGGCCAGC-3′ for Δ119–122, and 5′-GGCCAGCGCTGCTTACTCTCGCTCCTTC-3′ for S127A + T128A. Products from the second PCR were inserted into the pmaxCloning vector encoding a C-terminal V5 tag (see above) using *Bgl*II/*Bam*HI and *Xho*I restriction sites.

### 2.2 Cell culture, transfections and drug treatments

*Theileria annulata* (TaC12) and *T. parva* (Muguga)-infected cells were cultured in Leibovitz 15 medium (Gibco) supplemented with 10% fetal calf serum (FCS, Amimed), 10 mM Hepes pH 7.2 (Merck), 2 mM l-glutamine (Gibco), 70 μM β-mercaptoethanol (Merck), and antibiotics (Lonza). SV40-transformed cell lines of *Theileria*-uninfected bovine macrophages (BoMac) and monkey COS-7 cells, as well as HeLa cells were cultured in DMEM medium (Gibco) supplemented with 10% FCS and antibiotics. Transfections were done using Lipofectamine 2000 (Invitrogen) or Amaxa Nucleofection Kit V (BoMac) and R (COS-7) following the manufacturer's instructions. For elimination of the parasite, cultures of *Theileria annulata*-infected TaC12 cells were cultivated in the presence of 50 ng/ml of the theilericidal drug BW720c (Pitman-Moore). To induce merogony, TaC12 cells were treated for 8 days with 50 M chloramphenicol (Sigma) [16].

### 2.3 Microscopy and antibodies

Cells were grown on coverslips or poly-l-lysine coated coverslips (Sigma) and fixed with 4% paraformaldehyde in PBS for 10 min at room temperature or with methanol for 5 min at −20 °C. Cells were then permeabilized in 0.2% Triton X-100 prepared in PBS for 10 min at room temperature. Antibody incubations were done in PBS containing 10% FCS. DNA was stained with DAPI (Molecular Probes) and samples were mounted in Glycergel (Dako). Images were acquired on a Nikon Eclipse 80i fluorescence microscope equipped with a Retiga 2000R CCD camera (QImaging) using 40×, 60× and 100× Plan Apo objectives (Nikon) and Openlab 5 software (Improvision). Images were processed in Photoshop (Adobe).

Primary antibodies used in this study were: mouse monoclonal anti-Aurora B (AIM-1, clone 6, BD Transduction Laboratories), anti-cytochrome *C* (clone 7H8.2C12, BD PharMingen), IL-S40.2 (used in Fig. 2A), anti-PIM MAb5 (used in Fig. 2E and F; International Livestock Research Institute, Nairobi, Kenya), anti-α-tubulin (clone DM1A, Sigma), anti-V5 (Invitrogen), and 1C12 (detects *T. annulata* schizont surface; B. Shiels, University of Glasgow), as well as rabbit polyclonal anti-*T. parva* gp34, anti-TamR1 [19], and rat polyclonal anti-gp34. Appropriate secondary antibodies conjugated with fluorophores or horseradish peroxidase were used for IFM or western blot analysis, respectively.

To monitor cell division in COS-7 cells expressing EGFP-Tp-gp34, cells were transiently transfected (see above) and analysed by live imaging 4 h after transfection. Cells were recorded at 10 min intervals over 24 h at 37 °C and 5% CO_2_ using a TE2000E-PFS microscope (Nikon) equipped with a Plan Fluor 20× objective (Nikon), Orca ER CCD camera (Hamamatsu) and incubation chamber (Life Imaging Services). Movies were compressed using Quicktime software (Apple).

### 2.4 Triton X-114 phase separation and PI-PLC treatment

For surface expression of gp34, HeLa cells were transfected with plasmid encoding the complete coding sequence of TP01_0939 and epitope tags (see above). Transfected cells were cultured at 32 °C for 20 h before harvesting for either immunofluorescence analysis or PI-PLC treatment. For the latter, cells were harvested by trypsinization, washed twice in PBS, and resuspended in 10 mM Tris pH 7.5 and 150 mM NaCl. To these samples, PI-PLC from *Bacillus cereus* (Sigma) was added to a final concentration of 5 mU/μl and incubated at 37 °C for 1 h. Washed cell pellet and supernatant were finally analyzed by western blot.

Triton X-114 (TX114) phase separation was performed as described in [20]. Briefly, *Theileria*-parasitized cells (TaC12, Muguga) were lyzed in Triton X-114 extraction buffer (20 mM Tris pH 7.4, 100 mM NaCl, 20 mM β-glycerophosphate, 20 mM NaF, 1 mM Na_3_VO_4_, 1 mM EDTA, 1× Roche protease inhibitor cocktail, 1% TX114) for 1 h with frequent mixing. After centrifugation, lysate supernatants were incubated 30 min at 37 °C for phase separation. Samples were centrifuged again and the detergent phase was re-extracted twice with TX114-free buffer. Proteins of the primary aqueous phase and the detergent phase were concentrated by acetone precipitation and subjected to western blot analysis.

## 3 Results and discussion

### 3.1 Identification and expression of gp34

**Fig. 1:**
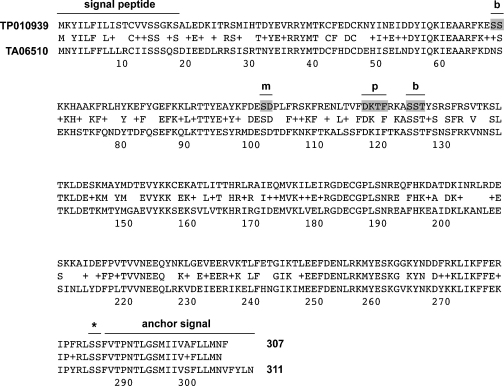
Sequence comparison of *T. parva* and *T. annulata* gp34. Pairwise alignment of *T. parva* and *T. annulata* gp34. The predicted signal peptide (SignalP 2.0) and GPI-anchor attachment signal sequence (DGPI 2.04) of both proteins including the GPI-anchor attachment site (asterisk) are indicated. Predicted motifs of *T. parva* gp34 are indictated: b, putative Plk1 Polo Box Domain (PBD) binding site; m, potential mimicking site for phosphorylation-primed PBD binding; p, putative Plk1 phosphorylation site; relevant residues are shaded in gray. Sequence positions showing residue similarity are marked by a plus sign in pairwise alignment.

A bioinformatics search of the predicted *T. parva* and *T. annulata* proteomes for GPI-anchored proteins expressed by the transforming schizont stage of both parasites yielded only few candidates. We focused on two hypothetical proteins XP_953835 (311 aa; TA06510) and XP_766460 (307 aa; TP01_0939), which display 63% identity and 81% similarity to each other. An alignment of the two proteins including the indication of a number of relevant motifs is shown in Fig. 1. This level of inter-species homology is well below the average percentage protein identity of 83.7% [15]. It is also lower than that of the two adjacent predicted proteins (TP01_0938/TA06505 and TP01_0940/TA06515) suggesting that TA06510 and TP01_0939 might be either under immune selection or, alternatively, play a role in species-specific host cell adaptation [15,21]. The two genes contain no introns and appear to be unique to *Theileria*, as a general Blast search, including other closely related *Apicomplexa*, did not reveal any orthologues. Massively parallel serial sequencing of *T. parva* schizont RNA [22] revealed low numbers of Tp-gp34 transcript (106 transcripts per million), a level similar to *Theileria rab* genes [23]. Even though genes encoding schizont GPI-anchored membrane proteins were reported to have low d*N*/d*S* values similar to house-keeping genes [15], with a d*N*/d*S* ratio of 0.163, gp34-encoding genes deviate from this pattern, potentially reflecting a certain degree of diversification in their regulatory functions that occurred after speciation of *T. annulata* and *T. parva*, or exposure to the immune system.

**Fig. 2:**
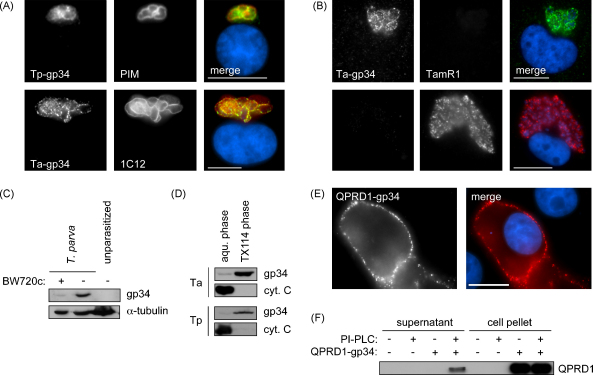
Characterization of gp34 a GPI-anchored protein expressed specifically by *Theileria* schizonts. (A) gp34 is expressed at the surface of the schizont. Immunofluorescence microscopy of *T. parva*- and *T. annulata*-transformed cells was carried out using antibodies directed against Tp-gp34 and Ta-gp34; the mAbs anti-PIM and 1C12 were used as markers for the parasite surface; DNA was stained using DAPI (blue); ‘merge’ represents an overlay of the three images; the scale bar represents 10 μm. (B) gp34 expression is downregulated during merogony. To induce merogony, *T. annulata*-infected TaC12 cells were cultured for 8 days in the presence of 50 μM chloramphenicol. The upper panels show a gp34-expressing schizont that had not yet started differentiation and is negative for the differentiation marker TamR1. The parasite depicted in the lower panels expresses TamR1, but has ceased to express gp34; DNA was stained using DAPI (blue); scale bar represents 10 μm. (C) Western blot analysis using anti-Tp-gp34 of cell lysates prepared from *T. parva*-transformed T cells, T cells from which the parasite has been eliminated by treatment with the specific theilericidal drug BW720c for 4 days, or unparasitized BoMac cells; α-tubulin was used as loading control. (D) Western blot analyses using anti-gp34 antibodies of *T. parva*- and *T. annulata*-transformed leukcoytes subjected to Triton X-114 extraction and phase separation (aqueous phase and TX-114 phase). Blots were probed for cytochrome *C* to confirm differential extraction. (E) QPRD1-gp34 expression on the surface of HeLa cells transiently transfected with a plasmid encoding a Tp-gp34 fusion protein consisting of the Tp-gp34 N-terminal signal peptide, part of the PIM QP-rich domain (QPRD1) carrying a PIM-specific epitope, and the gp34 core protein plus the C-terminal GPI anchor attachment signal. Surface-expressed protein was detected by IFM using a mAb directed against QPRD1. The cell nucleus was stained with DAPI (blue); scale bar represents 20 μm. (F) Western blot analysis of QPRD1-gp34 released into the supernatant upon treatment of transiently transfected HeLa cells (see E) with phosphatidylinositol-specific phospholipase C (PI-PLC). Untransfected HeLa cells (−) were used as control; ‘cell pellet’ shows cell-associated QPRD1-gp34 in cultures of transfected (+) or untransfected (−) HeLa cells, exposed or not exposed to PI-PLC.

The coding sequences were amplified by PCR and cloned into bacterial and mammalian expression vectors. Due to their apparent molecular mass in SDS-PAGE, the proteins were designated Ta-gp34 and Tp-gp34. Immunofluorescence microscopy (IFM) analysis using antibodies raised against both proteins confirmed that gp34 is expressed on the surface of *T. parva* and *T. annulata* schizonts (Fig. 2A). In both cases, a punctate staining pattern could be observed that differed from the smooth surface staining observed using anti-PIM or 1C12, a monoclonal antibody directed against another, uncharacterised, schizont surface protein [24]. Because of their flat shape, this pattern is best observed in *T. annulata*-transformed macrophages (Fig. 2A).

The capacity to transform parasitized cells is restricted to the schizont stage of *Theileria* and is lost when the schizont differentiates to the merozoite stage [25]. Using IFM, we investigated gp34 expression by parasites induced to undergo merogony by treatment with chloramphenicol [16]. The abundance of gp34 on the parasite surface correlated inversely with the expression level of the merozoite differentiation marker TamR1 and parasites showing high levels of TamR1 were negative for gp34 (Fig. 2B). Based on these observations we propose that expression of gp34 in the mammalian part of the parasite life cycle is restricted to the transforming schizont stage.

Western blot analysis of whole cell lysates prepared from *T. parva*-infected T cells (TpM(D409)T4) or *T. annulata*-infected macrophages (TaC12) (not shown) revealed proteins with an apparent molecular mass of 34 kDa (Fig. 2C). The protein was not found in extracts prepared from an (unparasitized) BoMac cell line and gp34 could hardly be detected upon elimination of the parasite by treatment with the theilericidal drug BW720c, confirming its parasite origin. Typical of GPI-anchored proteins, upon extraction with Triton X-114, both Ta- and Tp-gp34 partitioned into the detergent phase, whereas the hydrophilic marker protein, cytochrome *C*, remained in the aqueous phase (Fig. 2D).

We next tested whether the gp34 signal peptide and GPI-anchor signal are also functional in higher eukaryotes. To facilitate detection, a fragment of the PIM QP-rich domain (QPRD1) that harbours an epitope detected by MAb5 [11] was inserted immediately downstream of the predicted gp34 signal peptide sequence. Transiently expressed in HeLa cells, QPRD1-gp34 could be detected at the plasma membrane (Fig. 2E), showing the punctate pattern that was also observed in schizonts (see Fig. 2A). To verify if ectopically expressed QPRD1-gp34 was indeed GPI-anchored, cells were harvested and incubated with phosphatidylinositol-specific phospholipase C, which releases GPI-anchored proteins from the plasma membrane, while cells remain viable [26]. Treatment with PI-PLC led to a release of gp34 into the culture supernatant (Fig. 2F), confirming the protein was GPI-anchored.

### 3.2 Expression and localisation of gp34 in mammalian cells

Due to the absence of a surrounding parasitophorous vacuole, gp34, expressed on the surface of the *Theileria* schizont, is optimally positioned to interact with host cell structures. To investigate if gp34 has the capacity to bind to specific host cell structures, we transiently expressed Tp-gp34 or Ta-gp34 (shown for Tp-gp34) as soluble V5- or GFP-tagged proteins in COS-7 cells. To ensure expression in the cytosol, the constructs were designed to lack signal peptide and GPI anchor attachment signal sequences. In interphase cells, tagged Tp-gp34 and Ta-gp34 were routinely found in both the cytosol and the nucleus of transfected cells. The reason why ectopically expressed gp34 is also found in the nucleus is not clear, as the protein does not possess any obvious nuclear localisation signals. One possibility is that gp34, when expressed in the cytoplasm as a soluble protein, enters the nucleus by the (potentially fortuitous) interaction with a host cell protein that accumulates in the nucleus. Whichever, immunofluorescence analysis shows that parasite-expressed gp34 is normally tethered to the schizont surface and we presently have no microscopic evidence for nuclear translocation of gp34 in *Theileria*-transformed cells. The relevance of nuclear translocation observed for ectopically expressed gp34 must therefore be interpreted with caution.

**Fig. 3:**
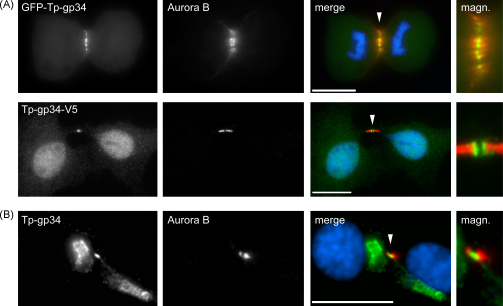
Tp-gp34 interaction with the central spindle and midbody. (A) IFM of COS-7 cells transiently expressing V5- or GFP-tagged forms of Tp-gp34 (green); the signal peptide and GPI anchor attachment signal were removed to guarantee cytosolic expression. The central spindle (upper panels; cell in anaphase) and midbody (lower panels; cell finishing cytokinesis) were stained using anti-Aurora B (red). DNA was stained with DAPI (blue). In the lower panels, ectopically expressed gp-34 can be seen accumulating in the nucleus; magn. = magnified image of the central spindle and midbody; the scale bar represents 20 μm. (B) IFM analysis using anti-Tp-gp34 (green) showing a representative example of *T. parva*-transformed cells in the process of completing cytokinesis; the midbody is visualized using anti-Aurora B (red); the arrowhead points at Tp-gp34 accumulation at the midbody; magn. = magnified midbody; scale bar represents 10 μm.

In COS-7 cells that had completed mitosis, GFP-Tp-gp34 and Tp-gp34-V5 were both found to localize to the central spindle and midbody (Fig. 3A). Central spindles are formed in the spindle midzone during sister chromatid separation and are compacted during the final stages of cell division to form the midbody that resides in the cytoplasmic bridge between the dividing daughter cells. Intriguingly, in contrast to Tp-gp34, V5- or GFP-tagged Ta-gp34 failed to interact with these structures (not shown). Similar observations were made when *Theileria*-transformed cells were stained for endogenous gp34. In dividing *T. parva*-infected T cells, gp34 was often found concentrated at the midbody (Fig. 3B), whereas no such accumulation could be observed in dividing *T. annulata*-infected macrophages (not shown). Midbody staining was not due to spurious cross-reactivity, as, in control experiments using unparasitized BoMac cells, anti-Tp-gp34 did not label midbodies (not shown).

Thus, despite the fact that Ta-gp34 and Tp-gp34 show many similarities, they also show some differences in their capacity to interact with host cell structures. The exact reason for this discrepancy is not yet known and may be based on differences between the two proteins in specific binding- or phosphorylation-motifs required for interaction with central spindle- or midbody-specific components.

### 3.3 Truncation/mutation analysis of Tp-gp34 expressed in mammalian cells

**Fig. 4:**
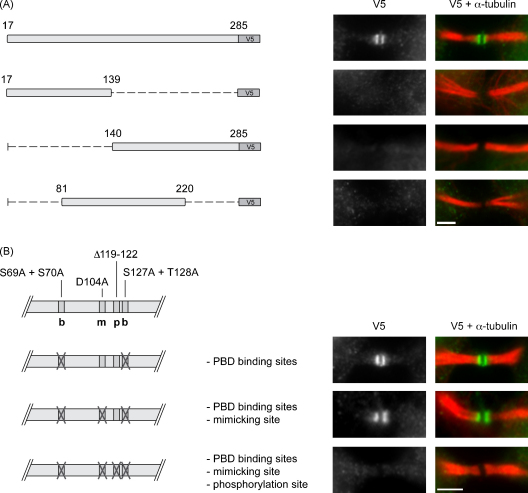
Truncation/mutation analysis of Tp-gp34 localisation to central spindle/midbody in COS-7 cells (A). COS-7 cells were transfected with plasmid constructs encoding full-length V5-tagged Tp-gp34 (excluding the signal peptide and GPI-anchor attachment signal) or truncation mutants spanning the N-terminal half (aa 17–139), the C-terminal half (aa 140–285) or the central region (aa 81–220) of Tp-gp34. Cells were stained for α-tubulin (red) to identify the midbody and V5 (green) for Tp-gp34 expression. Representative examples of midbody localisation are shown on the right. (B) The top panel shows a scheme of potential Plk1-interaction motifs in Tp-gp34 including the PBD binding sites (b), a mimicking site for PBD binding (m) and a Plk1 phosphorylation site (p); the corresponding aa substitutions and eliminations are indicated. COS-7 cells were transfected with plasmid constructs encoding mutagenized versions of full-length V5-tagged Tp-gp34 (aa 17–285) as indicated. Cells were stained and analysed as described for (A); scale bars represent 3 μm.

Next we carried out a more detailed analysis of Tp-gp34 to identify domains or motifs required for central spindle and midbody localisation. A range of different constructs, tagged C-terminally with a V5 epitope, were expressed in COS-7 cells and localisation monitored by IFM. Results presented in Fig. 4 shown that neither the N-terminal nor the C-terminal half of Tp-gp34 was capable of associating with the central spindle/midbody and the same applied to a construct spanning the middle region of the protein (aa 81–220). These data indicate that expression of a full-length Tp-gp34 is required to guarantee localisation to the central spindle/midbody in transfected COS-7 cells. Plk1 binding and phosphorylation have been shown to play an important role in targeting proteins to the central spindle/midbody [27]. We therefore tested a number of candidate motifs in Tp-gp34 that could potentially participate in this process. These included two putative Plk1 binding sites [28], a putative Plk1 phosphorylation site [29], as well as a site that could potentially mimic a PBD binding site (see Fig. 4B). An analysis involving the progressive elimination of these sites clearly showed that the phosphorylation site (aa 119–122, DKTF) was essential for targeting Tp-gp34 to the central spindle/midbody as perturbation of this site completely abrogated central spindle/midbody localisation. The absence of this phosphorylation site in Ta-gp34 (DKIF rather than DKTF) could help explain why Ta-gp34 fails tot localise to these structures when expressed in COS-7 cells. Thus, the interaction of Tp-gp34 with the central spindle/midbody in COS-7 cells is dependent on the protein being full-length (without predicted signal peptide and GPI anchor signal) and the presence of a putative Plk1 phosphorylation site. While interesting in its own right, it remains to be demonstrated that this site is indeed phosphorylated and also the biological significance of this localisation must be interpreted with care, in particular as Ta-gp34 does not show these characteristics in COS-7 cells.

### 3.4 Overexpresssion of gp34 induces binucleation in mammalian cells

**Fig. 5:**
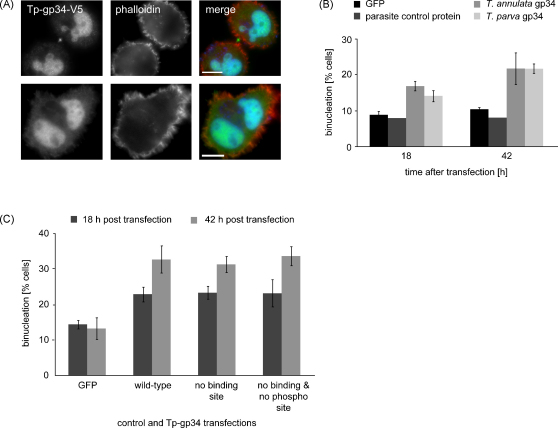
Overexpression of gp34-V5 induces cytokinetic defects. (A) Overexpression of Tp-gp34 in TaC12 cells provokes binucleation (lower panels), or nuclear abnormalities (upper panels) in dividing cells; nuclear accumulation of Tp-gp34-V5 and midbody staining is prominent; cell boundaries were visualized by phalloidin staining (red); DNA was stained with DAPI (blue); scale bars represent 10 μm. (B) At 18 or 42 h after transfection, the number of binucleated cells was monitored in BoMac cells overexpressing either GFP, a parasite control protein or gp34 from either species. Data represent the mean of four experiments with *n* = 40–300 cells/sample; error bars indicate standard deviations. (C) To test if the putative Plk1 motifs described in Figs. 1 and 4 are important for inducing binucleation, BoMac cells were transfected with constructs encoding wild-type Tp-gp34 (aa 17–285), a mutant form lacking all possible PBD binding sites, or a form of Tp-gp34 lacking all binding sites as well as the Plk1 phosphorylation site. Transfected cells were analyzed by IFM after 18 and 42 h and quantified for binucleation. GFP expression was used as negative control. Data represent the mean of three repetitions from two experiments with *n* = 100–200 cells/sample; error bars indicate standard deviations.

While examining cultures of cells transiently expressing gp34, we frequently observed binucleate cells or cells containing abnormal nuclear morphology indicative of a potential interference with mitosis/cytokinesis (Fig. 5A). To analyse this observation in more detail, V5-tagged Ta-gp34 or Tp-gp34 were transiently overexpressed in the cytosol of *T. annulata*-transformed TaC12 cells or BoMac cells. Mitosis and cytokinesis of transfected, V5-positive, cells were closely monitored by IFM and the number of V5-positive cells containing more than one nucleus was recorded. Overexpression of gp34 from either species did not affect mitotic events such as spindle formation, spindle maintenance or the formation of a metaphase plate. The process of cytokinesis, however, appeared to be disturbed, resulting in a significant increase in the number of binucleate BoMac cells (Fig. 5B). Similar effects could be observed in transiently transfected *T. annulata*-transformed TaC12 cells (data not shown). In this case, a 3-fold increase (from 8 to 25%) in the number of binucleate cells was observed over a period of 36 h.

We also tested Tp-gp34 mutants that lacked putative PBD binding sites, as well as a mutant in which both the PBD binding sites and the putative Plk1 phosphorylation site were disrupted. Interestingly, also in the latter case the number of binucleate cells increased significantly in gp34-expresssing cells compared to control cells.

We further used live video imaging to monitor cell division in BoMac cells expressing GFP-labelled gp34. Live imaging revealed an interference with furrow ingression and/or progression to full abscission. In a number of cases, cells entered mitosis, progressed to metaphase, but no obvious anaphase and furrow ingression could be observed (movie S1). In other cells, ingression started but was subsequently interrupted and furrow regression and the formation of binuclear cells could be observed (movie S2).

Thus, whereas gp34 overexpression in mammalian cells can clearly interfere with cytokinesis/abscission, a link between these findings and the localisation of gp34 to the central spindle/midbody observed in transfected COS-7 cells is not obvious, in particular as Ta-gp34, while perfectly capable of affecting cytokinesis, does not localise to these structures. The reason for this discrepancy is not yet known. Cytokinesis is a highly intricate process, subjected to multiple layers of regulation and involving the precise spatio-temporal activation of key regulator proteins [27]. Exactly where ectopically expressed gp34 interferes with this process remains to be elucidated.

## 4 Concluding remark

GPI-anchored proteins are usually expressed on the surface of cells, where they interact with the extracellular environment. Expressed on the surface of the *Theileria* schizont, gp34 is special in that it faces directly into the cytosol of another eukaryotic cell. The function of gp34 is not yet understood. The fact that no orthologues of gp34 were found in any other organism supports the concept that gp34 has a unique function in the biology of *Theileria*. To maintain the transformed state, the schizont must be partitioned over the two daughter cells at each host cell division. This process is important for parasite persistence. Whether gp34 participates in this process has not yet been established, but some of our findings indirectly suggest that gp34 could be involved. In particular the striking interference with cytokinesis/abscission by ectopically expressed gp34 could reflect the capacity of parasite-expressed gp34 to interact with key regulators of host cell cytokinesis. The physiological relevance of these observations, however, still needs to be confirmed. Experiments are presently underway to identify gp34-interacting partners of host cell origin. This, in turn might help elucidate the intricate interplay between the schizont and the host cell during mitosis.
