# Supplementary figures and images for: Characterisation of gp34, a GPI-anchored protein expressed by schizonts of Theileria parva and T. annulata
Source: Mol Biochem Parasitol. 2010 Aug;172(2):113–20. doi: 10.1016/j.molbiopara.2010.03.018 (PMC2880791; doi:10.1016/j.molbiopara.2010.03.018)

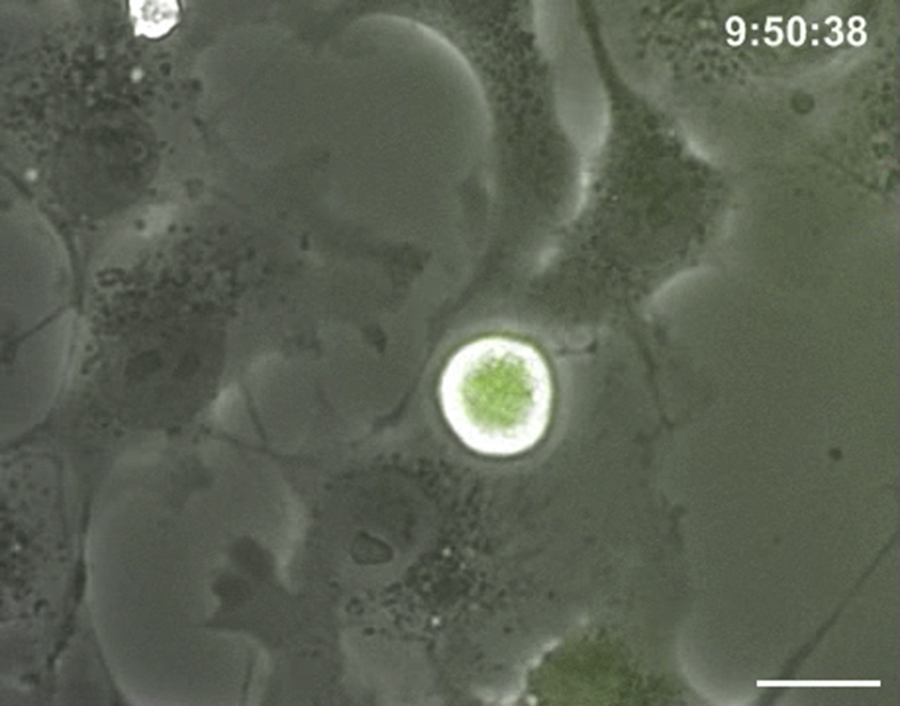

Supplement: Supplementary file 1 [file mmc1.jpg]

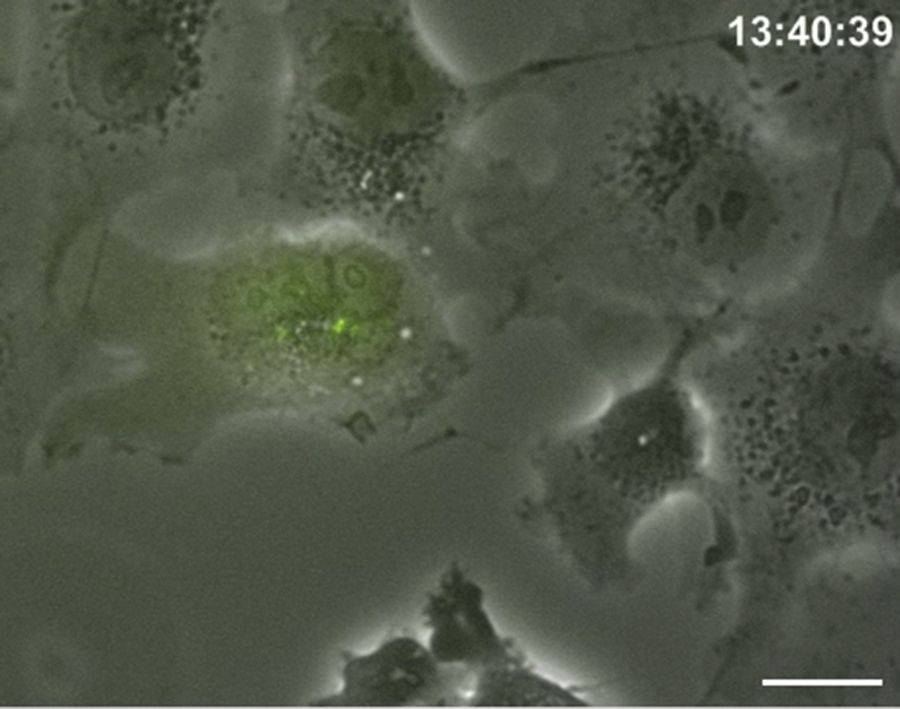

Supplement: Supplementary file 2 [file mmc2.jpg]
