# Supplementary material for: Characterisation of gp34, a GPI-anchored protein expressed by schizonts of Theileria parva and T. annulata
Source: Mol Biochem Parasitol. 2010 Aug;172(2):113–20. doi: 10.1016/j.molbiopara.2010.03.018 (PMC2880791; doi:10.1016/j.molbiopara.2010.03.018)
Supplement: Supplementary file 3 [file mmc3.doc]

**Movies S1 and S2. Cytokinetic defects upon EGFP-Tp-gp34 expression.**

Time-lapse movies of transiently transfected COS-7 cells expressing EGFP-Tp-gp34 (green). Time is shown as hh:mm:ss from the beginning of a 24 h observation period. Scale bars represent 20 μm.
